# The one-step preparation of green-emission carbon dots based on the deactivator-reducing reagent synergistic effect and the study on their luminescence mechanism†

**DOI:** 10.1039/c8ra03353f

**Published:** 2018-05-31

**Authors:** Chunjin Wei, Jun Li, Xincai Xiao, Tong Yue, Dan Zhao

**Affiliations:** School of Pharmaceutical Sciences, South-Central University for Nationalities Wuhan 430074 P. R. China wqzhdpai@163.com; National Demonstration Center for Experimental Ethnopharmacology Education (South-Central University for Nationalities) Wuhan 430074 P. R. China

## Abstract

The preparation of high-quality green-emission carbon dots (g-CDs) has always been the hot spot in the field of nano-materials. This paper reports the synthesis of high-quality g-CDs based on the synergistic effect between the deactivator (polyethyleneimine) and the reducing reagent (citric acid). The emission wavelength and the fluorescence intensity of the prepared CDs are adjustable by controlling the ratio of raw materials and other synthesis parameters, with quantum yields of 22.2% under optimal conditions. The g-CDs can be used as a fluorescent ink without any chemical modification. This paper further proposes the synthesis mechanism as well as the fluorescence mechanism of CDs based on the synergistic effect, laying the foundation of the synthesis and research of CDs.

## Abstract 2

This paper reports the synthesis of high-quality green-emission carbon dots based on the synergistic effect between the deactivator (polyethyleneimine) and the reducing reagent (citric acid).

## Graphical abstract

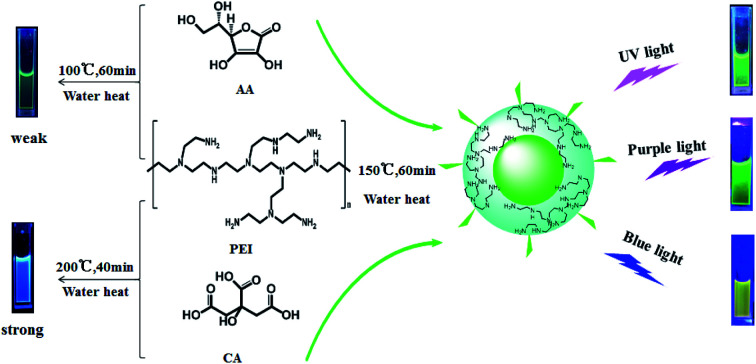

## 1. Introduction

Compared with traditional quantum dots and organic dyes, carbon dots (CDs) as a novel type of carbon nanomaterial, exhibit obvious merits, such as low toxicity, excellent biocompatibility, adjustable emission wavelength, easy feasibility to be functionalized, endowing them with a promising outlook in biomedicine, photoelectron, catalysis and sensing.^1–7^ The raw materials of CDs are abundant and easily available (*i.e.* glucose,^8^ citric acid (CA),^9^ candle soot,^10^*etc.*). Many methods have been reported for the preparation of CDs, but most prepared CDs emit blue fluorescence under ultraviolet excitation.^3,11–13^ Tan *et al.* has successfully used polyethyleneimine (PEI) and CA to prepare blue-emission CDs (b-CDs) with quantum yields (QYs) of 48% (*λ*_em_ ≈ 460 nm).^12^ However, the blue fluorescence of prepared CDs exhibits weak penetrating ability, and it can be easily interfered with by the self-luminescence of biological tissue. g-CDs or red-emission CDs (r-CDs), therefore, show great potential in practical applications to *in vivo* imaging,^14^ cancer curing,^15,16^ and light emitting diodes,^2^ and their preparation is thus the focus of the research.

There are a few reports on the synthesis of CDs (green to yellow emission), and their preparation is mainly achieved by surface modification^17^ or heteroatom doping,^18,19^ especially the selection of better raw materials.^20,21^ For example, Gu *et al.* used sugar as a carbon source and DEG as a reaction medium, and successfully prepared green-emission CDs (g-CDs) through a microwave method.^21^ Although the reaction time (1 min) is very short, the QY of the prepared CDs was only 2.4%. Lin and his team prepared N-doped multi-colored CDs by dissolving N-content aromatic compounds into organic reagent (120–180 °C, 12 h),^18,19^ and the prepared CDs exhibited strong QYs (g-CDs: 10.4%, b-CDs: 4.8% and r-CDs: 20.6%). However, the reaction time was quite long, and the application of organic reagents was not environment-friendly. The products need a complicated purification process, including high speed centrifugation and chromatographic separation. Hence, the simple and fast preparation of long wavelength-emission CDs is urgently needed in this field.

Ascorbic acid (AA) is an environment-friendly raw material for g-CDs,^20,22^ and the synthesized product is simple without further purification. Wu *et al.* has synthesized g-CDs with AA as carbon source, ethanediol and water as solvents (160 °C, 70 min), but the QYs of the products are only 6.79%, which are not appropriate for the application in biological or medical fields.^20^ Researchers then discovered that though the bare CDs is low in QYs, the proper modification would greatly improve their QYs.^8,20,23^ Zhang *et al.* acquired g-CDs with AA as carbon source, and KH791 as deactivator in the mixture of ethanol and water (92 °C, 12 h).^8^ They discovered that the presence of KH791 could improve QYs from 3.8% to 8.6%.

PEI is a typical water-soluble macromolecular polymer and an ideal material as deactivator in the preparation of CDs.^24,25^ Guo *et al.* prepared bare CDs with AA as carbon source in the mixture of ethanol and water (140 °C, 1 h), and then mixed them with PEI (70 °C, 3 h) to acquire PEI-modified CDs. The QYs of modified CDs (5.9%) is four times higher than that of bare ones.^25^ The improvement in QYs through PEI modification is affirmative, but the effect is limited.

Based on the previous studies, it is discovered that AA could work as the carbon source to prepare g-CDs, and its presence could improve the QYs of prepared CDs, but the improvement is limited. We proposed that the carbon source AA is reductive, prone to be destroyed in the process of preparation, especially with the presence of oxidant or in high temperature and pressure environment, which is unbeneficial to the formation of CDs. The introduction of reducing agent would stabilize AA. Synergistic reaction is great meaningful in the study of nano-materials, which could reduce the reaction time and temperature, improve the conversion ratio, reduce the side reactions and further improve the quality of the products.^26^ Some research teams have reported the preparation of CDs based on synergistic effect. For example, some researchers discovered that N–S-doped CDs exhibit higher QYs than N-doped or S-doped CDs.^27^

This paper reports the synthesis of high-quality g-CDs based on the synergistic effect between PEI (deactivator) and CA (reducing agent) with AA as the carbon source. Further studies have been carried out on the impacts of the ratio of reactants, reaction time and temperature upon the prepared CDs. Besides, the mechanisms of synthesis and photoluminescence are also explored through fluorescence spectra and IR spectra and fluorescence lifetime. The study is meaningful for the future studies on the synthesis and the mechanism of the g-CDs. Furthermore, the synthesized g-CDs have durable fluorescence, soluble in water very well and they can be used as a fluorescent ink without any chemical modification.

## 2. Experimental section

### 2.1. Chemicals

Polyethylenimine (PEI, *M*_w_ = 6 k, 8 k, 10 k, 99%) was purchased from Aladdin (Shanghai, China). Citric acid (CA, ≥99.5%), ascorbic acid (AA, ≥99.7%) and methanol (≥99.5%) were obtained from Sinopharm Chemical Reagent Co., Ltd. All chemicals used were of analytical grade or of the highest purity available. All solutions were prepared using Milli-Q water (Millipore) as the solvent.

### 2.2. Preparation of C-dots

The g-CDs_(AA–PEI–CA)_ were prepared as detailed in the following: 0.15 g (0.85 mmol) of AA, 0.18 g (0.1 mmol) of PEI and 0.8 g (3.8 mmol) of CA was dissolved in 10 mL of distilled H_2_O and the mixture was stirred at room temperature for 5 min. Then the solution was transferred to an autoclave for further reaction at 150 °C for 1 h.

For the purification of CDs_(AA–PEI–CA)_, methanol was added dropwise until the reaction mixture became turbid. Then the turbid dispersion was centrifuged (8000 rpm) for 10 min. Subsequently, the isolated CDs_(AA–PEI–CA)_ precipitate was dried under vacuum.

### 2.3. Characterization

UV-vis absorption spectra were acquired with a Lambda-35 UV-vis spectrophotometer (PerkinElmer Company). Fluorescence spectra were recorded on a LS55 spectrofluorometer (PerkinElmer Company). HRTEM images were obtained with a JEM-2100F transmission electron microscope (Japen Electron Optics Laboratory Company). Fourier transform infrared spectra were obtained on a Nicolet 6700 (IR) spectrometer (Thermo Fisher Scientific), and the sample was tested as solid powder. A FELIX32 system was used to obtain the fluorescence intensity decay curves (Photon Technology International, USA).

### 2.4. QYs calculations

The QYs of CDs_(AA–PEI–CA)_ and CDs_(AA–PEI)_ were calculated by comparing the integrated fluorescence intensities and absorbance values of the samples (exited at 420 nm), using rhodamine 6G as the standard (QYs = 95% in ethanol).^28^ The relative QYs can be calculated by using the below equation:
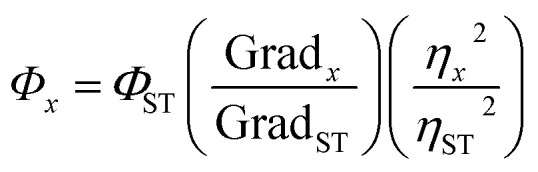
where *Φ* is the QYs, Grad is the gradient from the plot of integrated fluorescence intensity *versus* absorbance, and *η* is the refractive index (1.33 for water and 1.36 for ethanol); ST denotes the standard and *x* denotes the sample. To minimize reabsorption effects, absorption was always kept below 0.1 at the excitation wavelength.

Unless otherwise indicated, the conditions were as follows: AA: 0.15 g, PEI (*M*_w_ = 1800): 0.18 g, CA: 0.8 g, temperature: 150 °C, time: 60 min.

## 3. Results and discussion

### 3.1. The synthesis of CDs_(AA–PEI)_

**Fig. 1:**
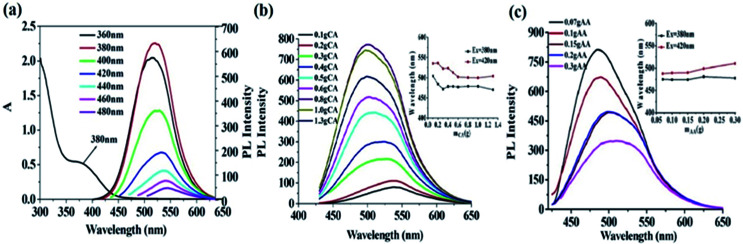
(a) UV-vis absorption spectra and emission spectra of CDs_(AA–PEI)_ obtained at the excitation wavelength of 360–480 nm with 20 nm increase (slit width: 10–15 nm); (b) impacts of different amount of CA on the fluorescence intensity of CDs_(AA–PEI–CA)_. The inset: the relationship between the amount of CA and emission wavelengths of CDs_(AA–PEI–CA)_ (*λ*_ex_ = 380 and 420 nm); (c) impacts of different amount of AA on the fluorescence intensity of CDs_(AA–PEI–CA)_. The inset: the relationship between the amount of AA and emission wavelengths of CDs_(AA–PEI–CA)_ (*λ*_ex_ = 380 and 420 nm).

Without the presence of reducing reagents, the g-CDs_(AA–PEI)_ were synthesized through hydrothermal route with AA as the carbon source and PEI as the deactivator under ideal conditions (AA:0.2 g, PEI (*M*_w_ = 1.8 k):0.18 g, pH = 5, 100 °C, 60 min). The QYs of prepared CDs was only 1.43% (*λ*_ex_ = 420 nm). The optimization process of reaction parameters was presented in ESI (Fig. S1–S5†). Fig. 1(a) shows the UV-vis absorption spectra and fluorescence spectra of prepared CDs. There exists an obvious absorption peak at 380 nm in the UV-vis spectra of CDs_(AA–PEI)_. When the excitation wavelength moves from 360 nm to 480 nm, the emission peak shifts from 511 nm to 541 nm with obvious decrease in fluorescence intensity.

Under the same reaction environment, the carbon source AA itself only produced clear solvent without color or fluorescence, showing AA alone could not prepare fluorescent CDs. The experiment results illustrate that the presence of the deactivator PEI plays a very important role in the synthesis of g-CDs. As a net-structured macromolecule rich in amino, PEI could combine with AA, and form g-CDs after the processes of dehydration and the growth of crystal nucleus. Although AA and PEI together could produce g-CDs, the QYs (1.43%) of the products are extremely low, and the adjustability is poor in the emission peak, making it unfit for the application in the fields of biology and medicine.

According to some related researches and our previous work, we believe that the reaction conditions have a significant effect on the properties of the prepared CDs. The carbon dots prepared at low temperature and pressure exhibited low QYs,^29^ while the hydrothermal route, due to its high reaction temperature and pressure, showed obvious merits like fast reaction rate, less surface defects, better crystallization, and less congregation, which could improve the fluorescence properties of prepared CDs.^30^ However, the experiments showed that the mere presence of PEI with AA would produce black CDs solvent with weak fluorescence under high temperature (140 °C) or longer reaction time (80 min) (ESI, Fig. S3 and S4†). It is proposed that the active AA exhibits excellent reducibility in the reaction, prone to be oxidized into dehydroascorbic acid and can be further hydrolyzed into diketogulonic acid under acidic condition (ESI, Fig. S6†). The oxidization process would be more violent under high temperature and pressure. This process destroyed the structure and active groups of AA, unbeneficial to the formation of carbon core and surface fluorophores, leading to the extremely low QYs of prepared CDs. To solve this problem, CA was introduced into the system as the reducing reagent to consume the produced oxidation materials during the process, and to enhance the stability of AA under such high temperature and pressure. The experiments show that the presence of CA is beneficial to the formation of high-quality g-CDs.

### 3.2. The synthesis of high quality g-CDs_(AA–PEI–CA)_

#### 3.2.1 The impact of CA upon the fluorescence property of CDs

The experiments show that the fluorescence of CDs is obviously improved with the presence of reducing reagent. The impact of CA amount upon the fluorescence properties of prepared CDs_(AA–PEI–CA)_ was investigated with other experiment parameters fixed (AA: 0.15 g, PEI_1800_: 0.18 g, 150 °C 60 min). As shown in Fig. 1(b), with the increase of the amount of CA, the fluorescence of prepared CDs first increases, and then decreases when the amount reaches 0.8 g. The excitation wavelength is correlated with the emission wavelength (inset of Fig. 1(b)). With the increase of CA, the emission wavelengths all shift to short-wavelength direction, either excited at 380 nm or 420 nm.

It can thus be concluded that the presence of CA in the system can effectively protect AA, beneficial to the synthesis of high-quality g-CDs. When the amount of CA is not enough to protect AA, the fluorescence of g-CDs is weak. The abundant amount of CA would ensure effective protection for the stability of AA, and is conducive to the preparation of high-quality g-CDs. However, the excessive amount of CA would react with PEI to form b-CDs (proven by the blue-shift of the emission peak of prepared CDs), causing insufficient amount of PEI for its reaction with AA to form g-CDs. The optimal CA amount used in the system was then set at 0.8 g.

#### 3.2.2 The impact of AA amount upon the fluorescence properties of CDs

With other reaction parameters fixed, the impact of AA amount upon the fluorescence properties of CDs was then examined. As shown in Fig. 1(c), with the increase of AA amount, in the range of 0.07 g–0.3 g, the fluorescence intensity of prepared CDs_(AA–PEI–CA)_ decreases gradually with the emission wavelength shifting to long-wavelength direction. Since the purpose of the experiments is to acquire g-CDs, taking the fluorescence intensity into consideration, we set AA at an optimal amount of 0.15 g. More interestingly, as shown in the inset of Fig. 1(c), when using 380 nm and 420 nm as the excitation wavelengths, the difference of emission wavelengths Δ*λ*_em_ (Δ*λ*_em_ = *λ*_em420_ − *λ*_em380_) gradually enlarges with the increase of AA amount.

It can be shown that the g-CDs synthesized by AA and PEI and the b-CDs formed by CA and PEI would superpose. When AA is insufficient, the g-CDs formed by AA and PEI were less than the b-CDs formed by relatively excessive CA and PEI. The overlapping of blue and green luminescence makes the emission wavelength short. With the increased amount AA, the produced g-CDs exceed b-CDs, resulting in the red-shifting of the emission wavelength. However, because the QYs of g-CDs is lower than those of b-CDs, and the excessive amount of AA would decompose without sufficient protection from CA under high temperature and pressure, the fluorescence intensity of prepared CDs gradually decreases. The acquired spectra are actually the mixed spectra of b-CDs prepared by CA and PEI and g-CDs prepared by AA and PEI.

#### 3.2.3 The impact of PEI upon the fluorescence property of CDs

**Fig. 2:**
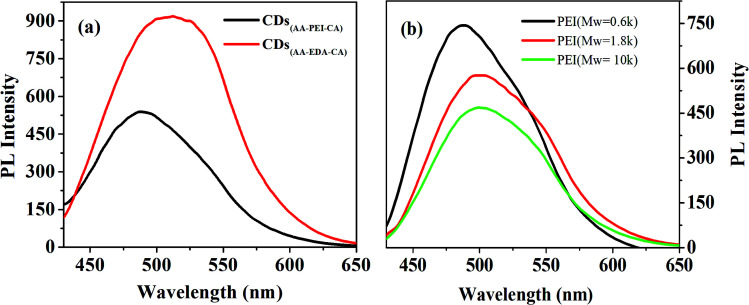
(a) impacts of different kinds of deactivators on the fluorescence intensity of CDs; (b) impacts of different molecular weight of PEI on the fluorescence intensity of CDs_(AA–PEI–CA)._

The previous experiments show that PEI is important for the synthesis of g-CDs. According to some researches, there are various deactivators, including polyethyleneglycol (PEG), PEI, ethylenediamine (EDA), and amino acid.^25,31–33^ With other experiment parameters fixed, PEI and EDA were used as deactivators in the system separately to investigate their effects on the fluorescence properties of the system. As shown in Fig. 2(a), both PEI and EDA could prepare fluorescent CDs, which are obviously better than the bare CDs made purely by AA, showing again the importance of deactivator in the system. However, the fluorescence intensity and the emission wavelength (*λ*_em_ ≈ 512 nm) of CDs prepared with PEI are better than those prepared with EDA (*λ*_em_ ≈ 488 nm).

The bare CDs would generally contain electron-withdrawing group, which would reduce the fluorescence of prepared CDs.^34^ The amino groups of the deactivator (PEI or EDA) could combine with the electron-withdrawing groups which are on the surface of the CDs, effectively reducing their amount of electron-withdrawing groups and thus improving the fluorescence intensity of prepared CDs. However, the small molecular weight, simple structure and the low degree of polymerization of EDA make it hard to combine with the carbon source to form fluorescent CDs. In contrast, PEI is a high molecular polymer rich in amino groups, and its high polymerization degree ensures the effective combination with AA to form carbon cores with large particle size, which is propitious to the preparation of long-wavelength g-CDs. Therefore, PEI is an ideal deactivator in the system.

PEI is a high molecular polymer that molecular weight greatly affects its degree of polymerization and viscosity.

The properties of CDs prepared by PEI with different molecular weights might be diverse. With other experiment parameters fixed, the effects of the molecular weight of PEI (*M*_w_ = 0.6 k, 1.8 k, 10 k) on the fluorescence properties of prepared CDs_(AA–PEI–CA)_ have been investigated. As shown in Fig. 2(b), the PEI with low molecular weight (*M*_w_ = 0.6 k) produces the CDs with strongest fluorescence intensity (QYs = 22.5%) and short wavelength, while the PEI with high molecular weight (*M*_w_ = 1.8 k) produces the CDs with weaker fluorescence intensity (QYs = 22.2%) and wavelength red-shifting 15 nm. Further increase in molecular weight (*M*_w_ = 10 k) would cause weaker intensity. So, the PEI with molecular weight at 1.8 k was then chosen for the system.

The molecular weight and the degree of polymerization of the deactivator are considered to have effects on the synthesis of CDs. EDA is not beneficial to the form of carbon core because of its low molecular weight, and PEI, due to its high molecular weight, is beneficial to that. However, PEI with the excessive high molecular weight shows excessive high congregation degree and bad water-dispersivity, preventing its sufficient contact with AA. Moreover, PEI with excessive high molecular weight would be serious carbonized in the experiment environment (high temperature and pressure), and the luminescence groups on the surface are easily destroyed, resulting in dark color of prepared CDs and weak fluorescence intensity.

With other experiment parameters fixed, the impact of PEI_1800_ amount upon the fluorescence properties of prepared CDs_(AA–PEI–CA)_ was examined. The experiment results are presented in ESI(Fig. S7†). The result shows that the amount of PEI_1800_ does not influence the emission wavelength of prepared CDs_(AA–PEI–CA)_, but the fluorescence intensity obviously. We consider that the little amount of PEI leads to uncompleted or thin modification layer, failing to fill up the surface defects of CDs and thus causing weak fluorescence intensity, while the excessive amount of PEI_1800_ would make AA disperse among different PEI_1800_ molecules, which reduce the amount of AA on single PEI molecule. Due to the repulsive forces between like charges among PEI_1800_ molecules, the dispersed situation of PEI_1800_–AA combinations is unbeneficial to the form of large-sized carbon cores and consequently the improvement in fluorescence intensity.

#### 3.2.4 The impacts of reaction temperature and time upon the synthesis of CDs

**Fig. 3:**
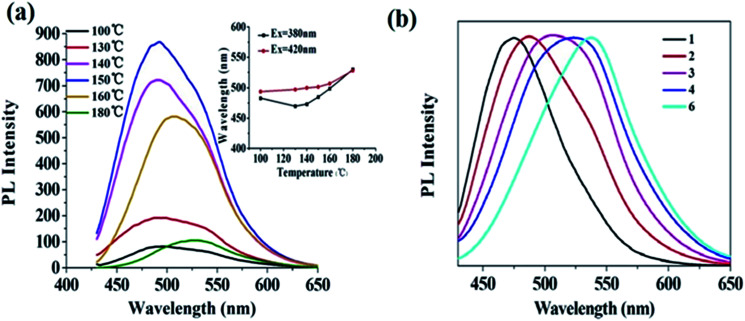
(a) Impacts of reaction temperature on the fluorescence intensity of CDs_(AA–PEI–CA)_. The inset: the relationship between the reaction temperature and emission wavelengths of CDs_(AA–PEI–CA)_ obtained at the excitation wavelength of 380 and 420 nm; (b) fluorescence spectra of CDs_(AA–PEI–CA)_ under different synthetic conditions (*λ*_ex_ = 420 nm).

The reaction temperature and time are important factors for the synthesis of CDs. Improper time and temperature would deter the formation of CDs.^12^ With other experiment parameters fixed, the impact of reaction temperature upon the fluorescence intensity of prepared CDs was then examined. As shown in Fig. 3(a), the fluorescence intensity enhances with the increase of reaction temperature, and reaches the peak at 150 °C (reaction time 60 min). It illustrates that high temperature is beneficial to the fast formation of carbon core through the dehydration and carbonization of the raw material, and the reduction of the surface defects of the prepared CDs, which can thus improve the fluorescence intensity.

As shown in Fig. 3(a), the reaction temperature has obvious effect on the emission wavelength of prepared CDs. With the increase of temperature, the emission wavelength is red-shifted. Interestingly, the emission wavelengths of prepared CDs excited by 380 nm and 420 nm get close with the increase of reaction temperature, showing that proper temperature (150 °C) is beneficial to the synthesis of high-quality g-CDs. However, when the temperature surpasses 160 °C, the color of the product turns dark with obvious decrease of fluorescence intensity. This may be the result of carbonization and decomposition of raw material before their interaction under high temperature, which prevents the formation of carbon core and destroys the luminescence groups on the surface of CDs and thus results in the sharp decrease of fluorescence intensity.

The impact of reaction time (40–80 min) upon the prepared CDs_(AA–PEI–CA)_ was also examined, and the experiment results are presented in the ESI (Fig. S8†). The result showed that the optimum reaction time is 60 min.

#### 3.2.5 The preparation of CDs_(AA–PEI–CA)_ with controlled emission wavelength

Through the study on the previous experiments, it is discovered that the amount of AA and CA, reaction time and temperature could not only impact the emission wavelength, but also the fluorescence intensity of prepared CDs, while the amount of deactivator PEI exhibits limited impact on the emission wavelength. Therefore, the emission wavelength of CDs can be adjusted by controlling of the synthesis parameters (474–537 nm, blue emission to yellow emission). Table 1 shows the emission wavelength of prepared CDs under different synthesis conditions, and Fig. 3(b) shows the fluorescence spectra of each CDs (the fluorescence intensity of no.5 CDs is too weak, and thus omitted in the figure).

**Table 1:** Synthesis conditions of CDs with Different Emission Wavelengths

Sample	*λ*_em_/nm	AA/g	CA/g	PEI_1800_/g	*t*/min	T/°C
1	474	—	0.8	0.18	60	150
2	486	0.1	0.8	0.18	60	150
3	504	0.15	0.8	0.18	60	150
4	512	0.15	0.5	0.18	60	150
5	521	0.15	—	0.18	60	150
6	537	0.2	0.2	0.18	60	150

### 3.3. The characterization of CDs_(AA–PEI–CA)_

#### 3.3.1 Fluorescence spectra

**Fig. 4:**
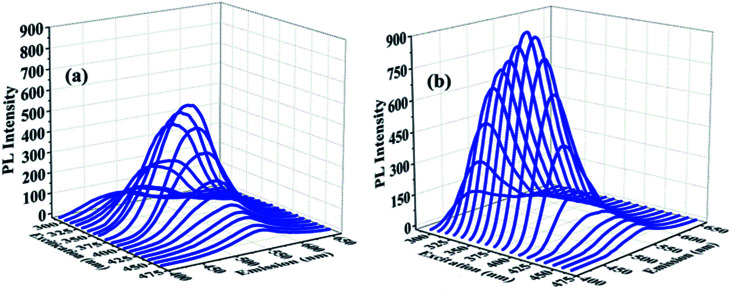
3D fluorescence spectra of CDs_(AA–PEI)_ (a) (slit width: 10–15) and CDs_(AA–PEI–CA)_ (b) (slit width: 10–10).

Fig. 4 shows the acquired CDs_(AA–PEI)_ and CDs_(AA–PEI–CA)_ three-dimensional (3D) fluorescence spectra by increasing the excitation wavelength scanned every 10 nm in the range of 300–480 nm. The 3D fluorescence spectra show that though the wavelength of CDs_(AA–PEI)_ and CDs_(AA–PEI–CA)_ both shift with the increase of excitation wavelength, there is a big difference between their spectra. The optimal excitation and emission wavelengths of CDs_(AA–PEI)_ are at 380 nm and 517 nm, while those of CDs_(AA–PEI–CA)_ are at 420 nm and 504 nm. CDs_(AA–PEI–CA)_ maintains strong fluorescence in the wide range of excitation wavelength (475–535 nm), while the effective wavelength for CDs_(AA–PEI_) is quite narrow (500–535 nm), and the fluorescence intensity sharply drops with the increase of excitation wavelength, which is obviously weaker than that of CDs_(AA–PEI–CA)_.

#### 3.3.2 TEM

**Fig. 5:**
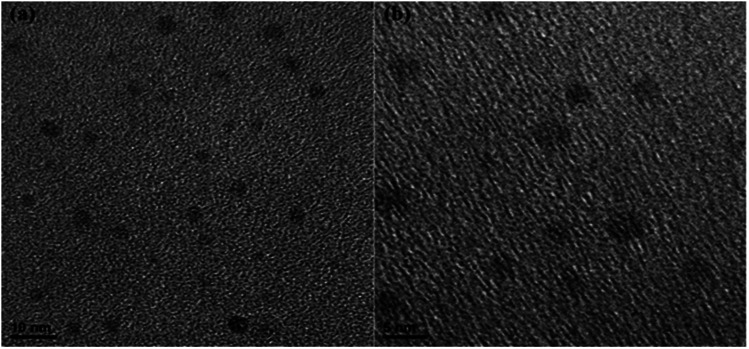
HRTEM images of CDs_(AA–PEI–CA)_.

TEM has been extensively utilized as a powerful tool to study the morphology of CDs from which the size can be identified. HRTEM images (Fig. 5) clearly reveal that the as-prepared CDs_(AA–PEI–CA)_ are spherical and monodisperse, and the average diameter of CDs was about 3.6 nm.

#### 3.3.3 IR spectra

The IR spectrum was used to analyze the surface structure of prepared CDs. As shown in Fig. S9,† IR spectra of CDs_(AA–PEI–CA)_, CDs_(PEI–CA)_ and CDs_(AA–PEI)_ were measured. The results show that CDs_(AA–PEI–CA)_ have many similar characteristic absorption bands to that of CDs_(PEI–CA)_ (O–H of 3424 cm^−1^, –CONH– of 1713 cm^−1^, C–H of 1401 cm^−1^ and C–O/C–O–C of 1215 cm^−1^), but significantly different from that of CDs_(AA–PEI)_. Based on the IR spectra, it can be inferred that the source of –CONH– is the interaction between COOH and NH_2_. The inconspicuous O–H peak in IR spectrum of CDs_(AA–PEI)_ shows the fully reaction of OH from AA with NH_2_, while the O–H group from CDs may come from OH of CA. The IR spectra prove that CA and PEI interact with AA through synergistic effect.

#### 3.3.4 Fluorescence lifetime

The decay fitting results are listed in Table 2 and the fluorescence decay curves of CDs_(AA–PEI–CA)_ and CDs_(AA–PEI)_ are presented in ESI (Fig. S10†). The results show that CDs_(AA–PEI–CA)_ has three fluorescence lifetimes (*τ*_1_, *τ*_2_, *τ*_3_) and CDs_(AA–PEI)_ has two fluorescence lifetimes (*τ*_1_, *τ*_2_). The average fluorescence lifetimes of CDs_(AA–PEI–CA)_ and CDs_(AA–PEI)_ are 4.349 ns and 2.914 ns, respectively. Compared with CDs_(AA–PEI)_, CDs_(AA–PEI–CA)_ has a longer fluorescence lifetime and enhanced fluorescence intensity.

**Table 2:** Fitting parameters of the corresponding fluorescence decay curve

Sample	*λ*_ex_ [nm]	*λ*_em_ [nm]	*τ*_1_ [ns]	*A*_1_ [%]	*τ*_2_ [ns]	*A*_2_ [%]	*τ*_3_ [ns]	*A*_3_ [%]	Ave.*τ* [ns]
CDs_(AA–PEI–CA)_	481	535	3.115	70.75	11.23	18.89	0.226	10.36	4.349
CDs_(PEI–CA)_	481	535	0.3446	21.4	3.614	78.6	—	—	2.914

### 3.4. Fluorescent ink

**Fig. 6:**
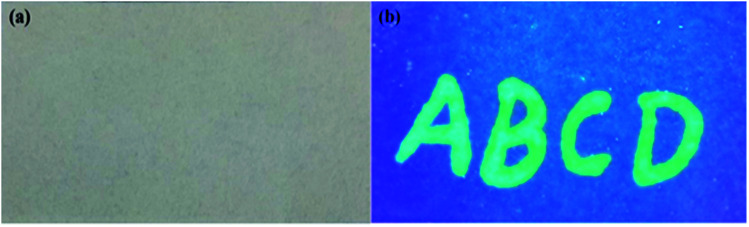
The fluorescent text is written on a filter paper under ambient light (a) and a UV-light with a wavelength of 365 nm (b) using g-CDs aqueous solution.

The g-CDs can be used as a new type of fluorescent ink due to their excellent dispersibility, solubility and strong fluorescence. The aqueous solution of g-CDs was directly injected into a pen without any chemical modification. Moreover, it was readily flow while writing without any leakage and coagulation within the pen. The handwriting on a filter paper was colorless under ambient light but showed bright green fluorescence under UV-light at a wavelength of 365 nm (Fig. 6). The “vis-invisible” and “UV-visible” properties make it possible for the application of g-CDs in anti-counterfeit fields. Therefore, the g-CDs ink might be an alternative and potential for fluorescent pens.^35^

### 3.5. The discussion of mechanisms

#### 3.5.1 The mechanism of synthesis

To further understand the synthesis mechanism of CDs_(AA–PEI–CA)_, the process of preparation was studied in detail. AA, CA and PEI were stirred at room temperature for 5 min and then the precursor solution was placed at a suitable temperature (150 °C) for a suitable reaction time (60 min) to obtain a high quality g-CDs (*λ*_ex_ = 420 nm, QYs = 22.2%). The CDs exhibit excitation wavelength-dependent fluorescence property and emit strong green fluorescence under the irradiation of UV light, yellow-green fluorescence under purple light (400–425 nm), and bright yellow fluorescence under blue light (465–475 nm).

**Fig. 7:**
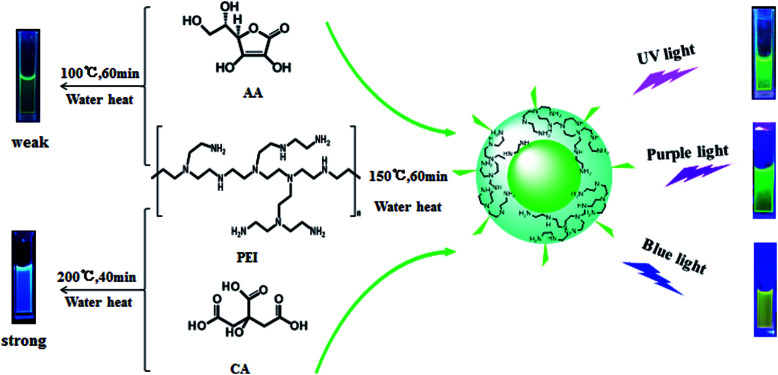
The possible synthesis mechanism of CDs_(AA–PEI–CA)_.

Fig. 7 shows the possible synthesis mechanism of CDs_(AA–PEI–CA)_, where AA works as carbon source, CA as the reducing reagent and PEI as the deactivator. Because of the synergistic effect between the deactivator and the reducing reagent, AA, CA and PEI can form a variety of organic luminescent groups under room temperature. The high temperature (150 °C) of the hydrothermal environment is beneficial to form more organic luminescent groups, and the existence of CA could prevent AA from decomposition, and then the hydroxyl, carboxyl and amino groups could fast aggregate and dehydrate to form carbon core.^9^ The existence of luminescent groups (amido bond) on the surface of CDs_(AA–PEI–CA)_ as shown in IR spectra (Fig. S9†) verifies the formation of fluorescence groups in the reaction. Furthermore, we hold that the deactivator PEI plays a significant role in the reaction: on the one hand, the branched structure of PEI can work as the backbone to combine with AA or CA, forming compact chain structure, which is beneficial to the process of dehydration and carbonization to form the carbon core; on the other hand, the amino group in PEI is prone to combine with the electron-withdrawing groups on the surface of CDs, reducing the surface defects and thus improving the fluorescence intensity of acquired CDs.^36^

The synergistic effect is also important in the synthesis of CDs_(AA–PEI–CA)_. Under the same reaction condition, the products of AA and CA exhibit no fluorescence, but PEI and CA produce CDs with blue fluorescence, and AA and PEI produce CDs with extreme weak green fluorescence (QYs = 1.43%). However, the presence of these three raw materials could produce g-CDs with high QYs (22.2%), showing the synergistic effect between the deactivator PEI and the reducing reagent CA.

#### 3.5.2 The mechanism of fluorescence

The fluorescence mechanism of CDs has always been a hot spot, but there is still lacking of consensus explanation to it.^9,36–39^ Yang *et al.*^39^ regarded the fluorescence of CDs under low temperature originates from the luminescence groups on the surface (like amide). At high temperature, the carbon core starts forming and the fluorescence of CDs originates from the synergistic effect between the carbon core and the surface luminescent groups, and the carbon core greatly affects the fluorescence of CDs with the increasing reaction temperature.^39^ Sun *et al.* consider that the fluorescence come from the radiative recombination of excitons on the surface energy traps of CDs. After the deactivation of the surface, these energy traps become stable, and thus turn to be fluorescence emissivity.^36^ However, according to the experiments, we propose that the fluorescence of CDs_(AA–PEI–CA)_ comes from two sources: the organic fluorescence groups and the carbon core. Since PEI is the macromolecular substance with branched structure, the hydroxyl (from AA and CA), carboxyl (from CA) and amino (from PEI) interact to form a variety of luminescent groups (mainly amide bond), which results in the weak green fluorescence in precursor solution. And the emission wavelength shifts with the movement of excitation wavelength.

The high-quality g-CDs_(AA–PEI–CA)_ (*λ*_ex_ = 420 nm, QYs = 22.2%) can be synthesized at a suitable temperature and reaction time. We consider that in the process of synthesis, the hydroxyl, carboxyl and amino would form large conjugated system polymers, which then dehydrate and carbonize under high pressure and temperature, and produce the carbon core capped by luminescent groups. As stated, the fluorescence of this structure originates from carbon core and the surface luminescent groups. Meanwhile, the synergistic effect between hydroxyl and amino group endows the prepared CDs excitation wavelength-dependent property. Under higher temperature (>150 °C) or longer reaction time (>60 min), the over-carbonization would make the product become dark or even black, with weak fluorescence, which shows that the high temperature environment would destroy the luminescent group on the surface of CDs, forming the carbon core without the capping of luminescent groups. Therefore, we believe that different synthesis conditions have a great influence on the structure and luminescence mechanism of prepared CDs.

## 4. Conclusions

This paper reports the preparation of g-CDs with AA as the carbon source, based on the synergistic effect of the deactivator (PEI) and the reducing reagent (CA). The as-prepared CDs exhibit strong luminescence and the QYs reach 22.2%. Moreover, the g-CDs can be applied as a new type of fluorescent ink. Importantly, through the comparison of CDs prepared by different raw material combinations, we propose the synthesis mechanism based on the synergistic effect between the deactivator and the reducing reagent. The results reveal that different conditions have great effects on the structure and luminescence mechanism of prepared CDs. The reported synthesis method is an ideal way for g-CDs for its simple operation, low temperature and short reaction, and the proposed mechanism offers inspiration for the future synthesis of CDs as well as the studies on them.

## Conflicts of interest

There are no conflicts to declare.

## Supplementary Material

RA-008-C8RA03353F-s001
