# Supplementary material for: The one-step preparation of green-emission carbon dots based on the deactivator-reducing reagent synergistic effect and the study on their luminescence mechanism
Source: RSC Adv. 2018 May 31;8(36):20016–24. doi: 10.1039/c8ra03353f (PMC9080747; doi:10.1039/c8ra03353f)
Supplement: RA-008-C8RA03353F-s001 [file RA-008-C8RA03353F-s001.pdf]

Electronic Supplementary Material (ESI) for RSC Advances.
This journal is © The Royal Society of Chemistry 2018

Supporting Information

The one-step preparation of green-emissioned carbon dots based on the deactivator-reducing reagent synergistic effect and the study on their luminescence mechanism

Chunjin Wei [a,b], Jun Li [a,b], Xincai Xiao [a,b], Tong Yue [a,b], Dan Zhao [a,b] *

[a] School of Pharmaceutical Sciences, South-Central University for Nationalities, Wuhan 430074, P. R. China

[b] National Demonstration Center for Experimental Ethnopharmacology Education (South-Central University for Nationalities), Wuhan 430074, P. R. China

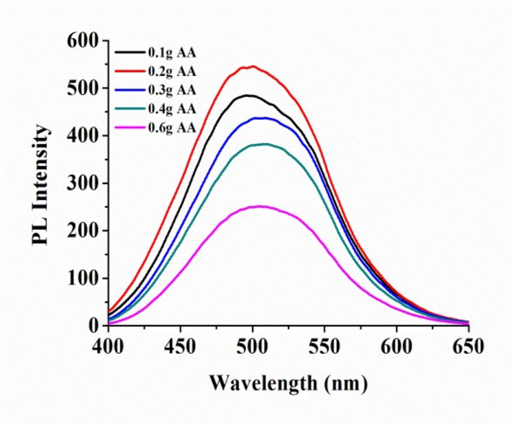

Fig. S1. Impacts of different amount of AA on the fluorescence intensity of CDs $_{(AA\text{-}PEI)}$.

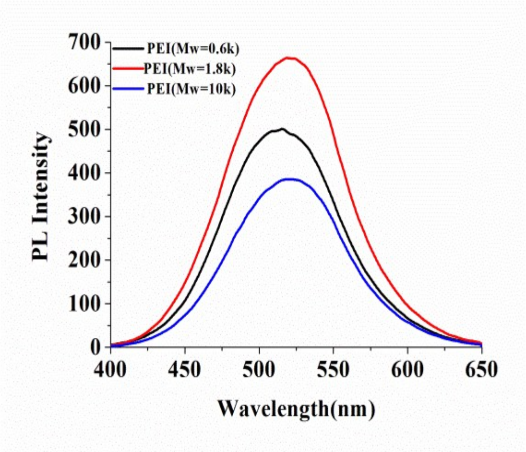

Fig. S2. Impacts of different molecular weight of PEI on the fluorescence intensity of CDs $_{(AA\text{-}PEI)}$.

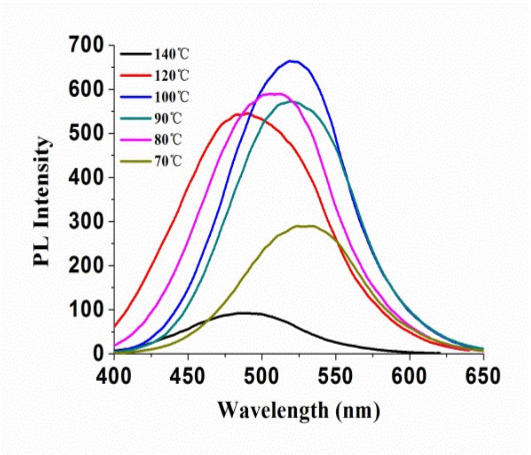

Fig. S3. Impacts of reaction temperature on the fluorescence intensity of $CDs_{(AA\text{-}PEI)}$.

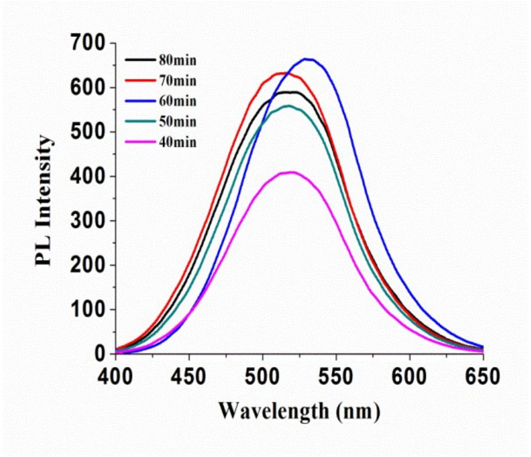

Fig. S4. Impacts of reaction time on the fluorescence intensity of $CDs_{(AA\text{-}PEI)}$.

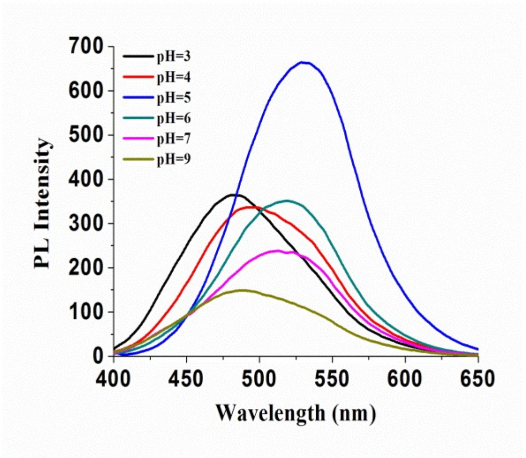

Fig. S5. Impacts of pH values on the fluorescence intensity of $CDs_{(AA\text{-}PEI)}$.

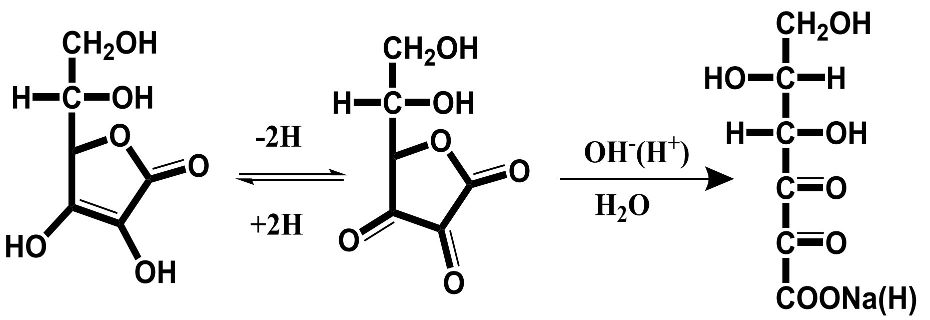

L- Ascorbic acid L-Dehydro ascorbic acid L-Diketogulonic acid

Fig. S6. The oxidation process of ascorbic acid.

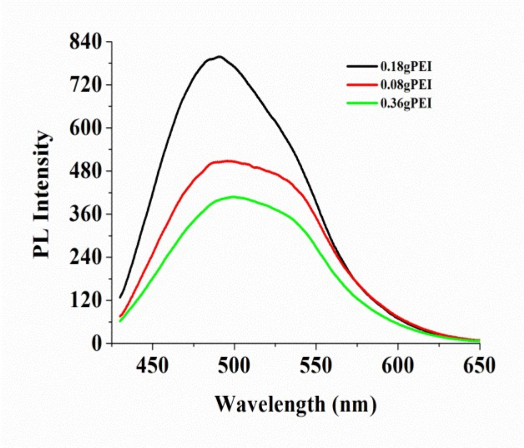

Fig. S7. Impacts of different amount of PEI on the fluorescence intensity of CDs（AA-PEI-CA）.

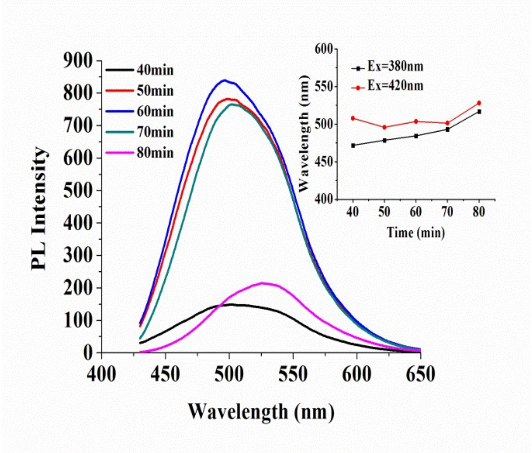

Fig. S8. Impacts of reaction time on the fluorescence intensity of CDs（AA-PEI-CA）.

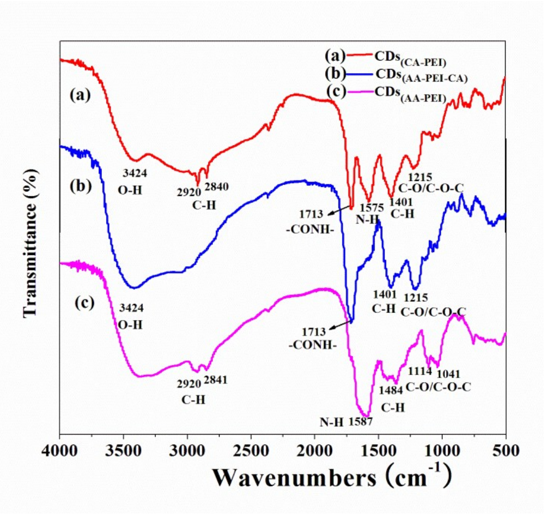

Fig. S9. IR spectra of $CDs_{(AA\text{-}PEI\text{-}CA)}$, $CDs_{(AA\text{-}PEI)}$ and $CDs_{(PEI\text{-}CA)}$.

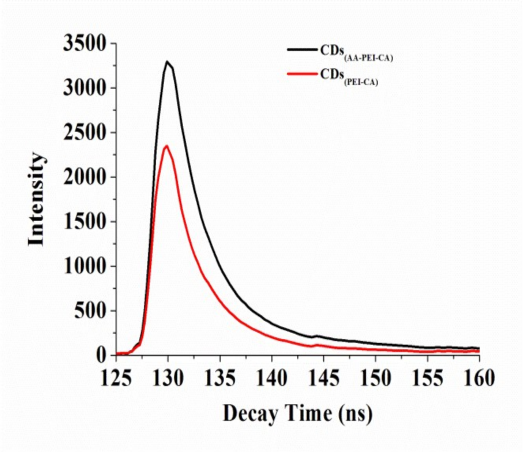

Fig. S10. Fluorescence decay curves of $CDs_{(AA\text{-}PEI\text{-}CA)}$ and $CDs_{(AA\text{-}PEI)}$.
